# Supplementary material for: Perceptual learning mechanisms with single-stimulus exposure
Source: PLoS One. 2026 May 8;21(5):e0327927. doi: 10.1371/journal.pone.0327927 (PMC13155592; doi:10.1371/journal.pone.0327927)
Supplement: Attachment — Submitted filename: The_Responses_To_Review.docx [file pone.0327927.s002.docx]

Dear Dr. Watson,

Thank you for the comments and opportunity to resubmit. We believe we have addressed every comment successfully. The response is not necessarily as long as it appears here (13 pages…) as we copy your letter and the reviews. Our responses to how we addressed the issues are marked with asterisks.

First, we would like to address the “Journal Requirements” that we are unsure of as the web submission form information does not appear to go through- or there is something about it we do not understand.

2. Thank you for stating the following in your Competing Interests section:

[No competing interest to declare].

Please complete your Competing Interests on the online submission form to state any Competing

Interests. If you have no competing interests, please state "The authors have declared that no

competing interests exist.", as detailed online in our guide for authors at

http://journals.plos.org/plosone/s/submit-now

This information should be included in your cover letter; we will change the online submission form on

your behalf.

**********************************************

We have No competing interests to declare.

3. When completing the data availability statement of the submission form, you indicated that you will

make your data available on acceptance. We strongly recommend all authors decide on a data sharing

plan before acceptance, as the process can be lengthy and hold up publication timelines.

**********************************************

In the web form for the prior submission, we indicated that the data are available without restriction, and provided a DOI and URL to them. These are also included in the revised manuscript.

8. Figure 1 in the manuscript is not copyrighted, and this was indicated in the prior submission. The figure was created by us from the game that we authored. It was created specifically for this manuscript and has not been published before.

Editor Comments: —————————————————————————————————————

In general the introduction should be more succinct, currently it is far too long and unwieldy. Please follow the reviewers advice on what can be left out and what should be included, I agree with these comments.

******************************************************************************

The introduction has been considerably trimmed (438 lines to 163), though one of the important factors regarding the work is that the effect of test stimulus exposure is not directly addressed in the theories around perceptual learning, so they did need some review.

As indicated by the reviewer, error bars should be included and within-subject error bars are particularly useful in this context. You can easily calculate within-subject error bars, see this link for a very useful step by step guide: https://www.cogsci.nl/blog/tutorials/156-an-easy-way-to-create- graphs-with-within-subject-error-bars

******************************************************************************

Error bars are now included. Cousineau’s method is simple, but we did not use it. Rather, we presented the standard error of the data. Though often incorrectly treated synonymously, error bars and hypothesis testing are different tools and we keep them as such here. Variation with within-subject differences removed, as derived from the analysis, is provided in the mean-squared error terms in the results, while between-subject variability around data points is presented in the figures.

It is a concern that colours were not fully counterbalanced - please explain this decision in the methods and acknowledge this as appropriate throughout the manuscript.

*****************************************************************************

We have elaborated on this slightly in the manuscript, but to do more, we would need to have more detail as to why that was a concern. Cue A was never compared to anything against which its identity should be counterbalanced (according to the concern, cue W in the retardation test should have been counterbalanced against something as well).

The effect of perceptual learning that the experiment concerns took place on elements B, C, and D (counterbalanced) when they were exposed in compound with other elements (X, Y, and Z, also counterbalanced). AX was used to train X in a compound to assess the effects of B (present originally with X) or not (D). “A” is irrelevant other than it was used to make a new compound with X and Y. Moreover there are practical issues. Counterbalancing A against the other stimuli which were being compared to each other would unnecessarily require 144 different stimulus combinations (elements1 A, B, C, D = 24; elements2 X, Y, Z = 6; elements1 x elements 2 = 6 x 24 = 144). If A should be counterbalanced for some reason that we are missing, so should W which is untenable.

1.	Review Comments to the Author

Reviewer #1: This study tests two explanations for the phenomenon that preexposure to a compound test stimulus (BX) alone suffices for learning (or reduces generalized aversion), after conditioning with another stimulus, AX. The mechanisms considered are retrospective reevaluation (RR) and restoration of perceptual effectiveness (RPE). Under RR, B comes to inhibits the outcome; under RPE, B becomes more salient or distinct from the conditioned compound. Two experiments were conducted using dichromatic symbol compounds in a video game, with suppression of a shooting response as the dependent measure. In both experiments, compounds BX and DX were pre-exposed, followed by conditioning with AX and AY. In Experiment 1,a summation test was conducted with compounds BY and DY, and BY passed the test. In Experiment 2,a retardation test was conducted with compounds BW and DW,and BW failed the test. The authors conclude that the results support RPE - or enhanced salience of B - as the learning mechanism.

This could be an interesting and useful paper provided some changes are made. Major comments

1.	Length and clarity of introduction: The introduction should be reduced substantially. At present, it reads more like the first draft of a master's thesis than a manuscript for publication. The phenomenon being studied and the competing hypotheses need to be explained earlier and more clearly and concisely. The purpose of the study should be stated in 4-5 pages total, focusing directly on: the effect of interest,the competing explanations (RR vs. RPE), whether either experiment is a replication of earlier work or whether both are novel. Other details - Gibson's ideas and mediated learning, for example - can be omitted.

*****************************************************************************

As discussed earlier above, the introduction has been considerably reduced.

2.	Relation to human perceptual learning literature: The study needs to be more clearly situated within the context of human perceptual learning, in addition to or instead of the animal learning studies. For example, Lavis et al (2011) showed that exposure to a single element can enhance discrimination.Is this similar to the effect reported here? If not, please clarify the differences. Mitchell & Hall (2014) provide a review of the issues raised here, and other work by Dwyer's group also should be cited.

*****************************************************************************

Lavis et al., (2011) did not show that exposure to a single element can enhance discrimination. They showed that exposure to both unique elements (A & B), in addition to intermixed exposure to AX/BX, enhanced discrimination. Following intermixed AX/BX exposure, compared to AX|BX (blocked), participants were presented with the shapes of A and B and were more accurate at selecting the color that they had been following the AX/BX schedule. They concluded that the AX/BX schedule produced better encoding of A and B.

In a second experiment they provided AX/BX exposure along with additional exposures of A and B intermixed in the trials. They argued that this additional exposure to the elements should produce more habituation of A and B, counteracting Hall’s restoration of perceptual effectiveness mechanism, and reduce the discrimination. On the other hand, these additional exposures should provide better encoding of A and B, enhancing the discrimination. The latter result was obtained. There was no independent assessment of whether the encoding was better with the color matching test used in experiment 1.

They appear to conclude that the intermixed/blocked effect is entirely explained by better encoding of unique features, when such better encoding may be encouraged by the intermixed exposure in addition to, or because of, the other mechanisms implicated (e.g., those of McLaren & Mackintosh). There is nothing to rule out that better encoding is an additional mechanism, as opposed to “the” mechanism. Assuming there are multiple mechanisms at play, whatever degradation of the Hall RPE mechanism that the exposure to the unique elements produced could be compensated for by the better encoding. Greater habituation of A and B could result in greater restoration of their salience through Hall’s mechanism just as well. These are just points to consider.

We have expanded the introduction and discussion to address more work done with humans and the ideas of Mitchell & his colleagues regarding enhanced encoding/attentional biases, but we do not get into much detail, nor do we mention the analysis above. In our experiment, all exposure to unique elements is equated, so all encoding/attentional biases are equal. The encoding hypothesis, as we understand it, predicts no effect whatsoever in our experiment.

The result we obtain is based on having experienced X’s associate again which, in theory, activated the representation of the absent B element. This treatment could have simply restored or enhanced the activation of that representation so that the next physical presentation of B was more effective, or enhanced its encoding by way of experiencing the retrieved representation. Either way, it seems that there is a better representation so there is a degree of isomorphism in the explanations. In that sense, the result is consistent with the spirit of both ideas and we mention that in the discussion. Multiple mechanisms undoubtedly play a role in the phenomena, and acknowledging that is important and enriches the understanding of things.

3.	Configural hypothesis (sections 2.1 and 2.2): The description of the configural hypothesis is not clear enough. Please state explicitly what predictions this hypothesis makes for each experiment, and how those predictions differ from the RR and RPE accounts. Please add all predictions to Table 1.

*****************************************************************************

We have modified our descriptions of the configural hypothesis, with additional references, to be shorter and to the point. We have not added predictions to the table, as they are simple (none of the theories discussed predict any effect) and now summarized in the text.

Stimulus salience: Cue A was always red, which is a particularly salient colour. This raises the question of whether the effects reported might have been different if A were less salient. The colour of A should have been counterbalanced across subjects. Please discuss this as a potential limitation of the results.

*****************************************************************************

As mentioned above, this statement needs clarification as the lack of counterbalancing A against the other cues, with which it was never compared, is irrelevant. The color of X does not affect the treatments or comparisons of the counterbalanced elements.

How the salience of A should matter or is a potential limitation is unclear. Apart from A not being involved in any comparison, and equally involved with counterbalanced elements in conditioning, it is questionable whether it was more salient. In phase 2 the training compound was Red and Green, Red and Brown, or Red and Orange. The salience of the red portion itself, and particularly in combination with the other colors, is likely a subjective matter of individual differences. We simply don’t see a relevant caveat here to discuss. Moreover, “the effects reported might have been different if A were less salient” without any explanation as to “why” is a “what if” criticism applicable to any aspect of the experiment (what if the elements were entire sensors and compounds were 2-sensor groups, what if sounds were used instead of colors, what if the chicken eggs were a different color) or any experiment ever published. We are certainly open to correction, but the concern seems invalid and, as a limitation, the choice of red here is no more limiting than any other choice made in the method.

Further comments: Abstract and General

The purpose of CY is unclear. If CY wasn't relevant to these findings,please remove it from the abstract and elsewhere.

*****************************************************************************

Y was the transfer excitor used in the summation test. X was the excitor used in the generalization test. To keep the two tests as comparable as possible, we equated X and Y. As X had been exposed in a compound, CY was used to expose Y in a compound.

Introduction:

-	Pages 10-11,lines 205-218: confusing and misplaced. If the results can be explained 'without appeal to configuration', why not say this earlier where you explain latent inhibition? Please clarify.

-	Page 11, line 223 onwards: confusing. Does blocking contradict the configural explanation? If not, why isn't the configural account sufficient?

-	Page 13, line 270 onwards: When AX and BX are exposed in alternation,you state that A and B's effectiveness is recovered. This seems the opposite of the mutual inhibition proposed by Mclaren. Please explain.

-	Page 15, lines 305 onwards: In the food+tone example,please add the compound stimulus notations (BX, AX etc) so as to compare with previous examples

-	Page 15 lines 314 onwards: from here onwards, I ceased to follow the logic. Please delete, or reduce and simplify.

*****************************************************************************

These issues have disappeared with the re-write of the introduction, with perhaps the exception of “Page 13, line 270 onwards: When AX and BX are exposed in alternation, you state that A and B's effectiveness is recovered. This seems the opposite of the mutual inhibition proposed by Mclaren. Please explain.” There is an apparent contradiction there, that might be resolved to an extent with a few assumptions, but they are not our theories to defend or reconcile.

Methods

-	The description of methods and the experiment (pages 18-28) is repetitive and could be shortened

*****************************************************************************

There is some repetition between the description of the experiment and the method. A few methodological details are necessary in the experiment description for the reader to understand the flow of events in the experiments and what is being manipulated. Those aspects should also be included in the method section, though in more detail. Within the method itself, we have carefully examined it for redundancy, we could identify little to remove.

-	Table 1 should include predictions for both experiments under RR, RPE, and the configural hypothesis

-

*****************************************************************************

These are simple predictions in that the pre-exposure was identical for all groups, thus the theories reviewed all predict no effect. We make these predictions clear at the end of each experiment’s description.

-	Page 20, line 426-428: The sentence ...better reducing suppression controlled by X by way of inhibition· is difficult to understand. Please rephrase.

*****************************************************************************

The sentence in question, “The restored effectiveness of B would make it functionally more salient and distracting on test relative to D, better reducing suppression controlled by X by way of external inhibition [55]” . We assumed readers would be familiar with Pavlov’s concept of external inhibition, but have now clarified it. The novel presence of an external very salient B relative to the novel presence of a less salient D simply disrupts ongoing suppression more, rather than B having an internal inhibitory association with an outcome.

Results

-	Please explain the suppression ratio measure in a bit more detail

*****************************************************************************

Computationally (ITI Rate / (CS rate + ITI rate), it cannot be expressed in more detail. The suppression ratio is a ratio of the response rate of the CS in question divided by the sum of the rates to the CS in question and the rate preceding the CS. It was developed in 1961 where it has routinely been used in studies, particularly conditioned emotional response “CER” work with rats leverpressing for food and receiving shock USs, on which the present method was conceptually modeled (clicking for points and receiving punishing egg attacks). We now include this information in the manuscript.

-	Figures 2-4. Error bars should be shown regardless of clutter. To reduce clutter, you could add them to the first and last symbols only. Or, you could plot within-subjects error bars (Cousineau, 2005), which would be appropriate here and typically smaller.

*****************************************************************************

Discussed above in the editor section.

-	Experiment 1a: Lines 644-655 say that passing the summation test indicates RE, but the discussion says it could indicate RE or RPE. Please clarify.

*****************************************************************************

We have clarified the text regarding passing summation testing and its implications throughout.

-	Experiment 1a: Please comment on performance differences between BX vs. BY. It appears suppression was different in those conditions. Does this have any implications?

*****************************************************************************

The ANOVA indicated two main effects, one of “Revaluation” indicating less suppression overall to compounds involving B than compounds involving D, and an effect of Seconds as suppression increased over the trial. There was no effect of whether the test was a generalization test (compounds involving X) or a summation test (compounds involving Y). There were no interactions. There is no evidence of a BX vs. BY performance differences to discuss.

-	Experiment 1b: The main effect of compound type is small, and likely emerges because of the power of the repeated-measures analysis. The discussion describes this as a clear effect, which is overstated. Please modify.

*****************************************************************************

“Clear” can be debated. The effect is clear in the analysis (e.g., no marginal p etc.), and our use of clear was more to interpretation rather than perceived size. That is, the interpretation of the main effect is not complicated by marginal probability or interactions. Nevertheless, we have removed “clear.”

Discussion

-	Expand the discussion of human perceptual learning studies (e.g., refs below)

-	Discuss the potential caveat of red A's salience

*****************************************************************************

Human work from the reviewer’s references are now discussed, but the caveat of red A’s salience is not. As discussed earlier, red may more salient, less salient, or equally salient across participants particularly when considering the counterbalanced stimuli with which it was presented in phase 2. Whether the effect depended on red is not relevant- the effect occurred. “red” may be a limiting condition, as many (if not all) effects have boundaries, but that is an entirely different experimental question based on a theory for why salience/color of the element with which the experimental elements were compounded in conditioning matters.

-	Page 34, line 709 onwards: The explanation of salience modulation is unclear. Please clarify

*****************************************************************************

The section has been modified to try to make it clearer. The idea is that the retrospective revaluation mechanism does not imply any change in perception, only a change in the associations tied to an element, where the restoration of perceptual effectiveness mechanism implies a change in perception. We also now point out how this idea fits with Mitchell’s ideas regarding encoding.

-	Page 35: This section is unclear and redundant with previous sections and not well tied to human learning studies

*****************************************************************************

We are unclear on the redundancy as this is the first place where we make the point that “conditioning” may not be necessary, only experiencing an associated element again. The point applies equally to human or animal studies. We do add that conditioning, as traditionally conceived, is absent in many human studies.

-	Page 36, lines 746-748: What is the evidence that •...differential latent inhibition...undoubtedly

contribute[s] to perceptual learning effects" in humans?

*****************************************************************************

The contribution of latent inhibition to perceptual learning phenomena is well known and documented. We now direct the reader to Bennett, C. H., Wills, S. J., Wells, J. O., & Mackintosh, N. J. (1994). Reduced generalization following preexposure: Latent inhibition of common elements or a difference in familiarity?. Journal of Experimental Psychology: Animal Behavior Processes, 20(3), 232-239 and Mackintosh, N. J., Kaye, H., & Bennett, C. H. (1991). Perceptual learning in flavour aversion conditioning. The Quarterly Journal of Experimental Psychology Section B, 43(3b), 297-322, following this statement.

The human work reviewed in the manuscript, as mentioned earlier, does not effectively rule out any of the mechanisms derived from animal learning. All use designs where things such as latent inhibition or inhibition between unique elements can operate, though they include manipulations to show that additional mechanisms (e.g., encoding of unique features) are in play. They do not rule out the operation of those other mechanisms. That is, the experiments are not designed where latent inhibition cannot operate, yet an effect is observed compared to groups where latent inhibition can operate and a greater effect is observed. We have modified the statement to “...should undoubtedly contribute to perceptual learning” as there are no experiments, we are aware of, that mirror the early work with animals assessing that mechanism with human participants.
