# Supplementary material for: Perceptual learning mechanisms with single-stimulus exposure
Source: PLoS One. 2026 May 8;21(5):e0327927. doi: 10.1371/journal.pone.0327927 (PMC13155592; doi:10.1371/journal.pone.0327927)
Supplement: Attachment — Submitted filename: ResponseToReview.docx [file pone.0327927.s003.docx]

**6. Review Comments to the Author

Please use the space provided to explain your answers to the questions above. You may also include additional comments for the author, including concerns about dual publication, research ethics, or publication ethics. (Please upload your review as an attachment if it exceeds 20,000 characters)**

**Reviewer #1: The revision of the manuscript is adequate and the authors have addressed most comments. A few remaining points:**

**1. Please correct grammatical errors and typos throughout. (e.g., line 87 promotes; line 100 awkward; line 190 that; line 542 might commanded, line 567 awkward clause at end of sentence; several more in discussion and other parts of the manuscript)**

We have addressed these issues by correcting typos and rewording. As well we found a few other minor grammar issues and changed a bit of the wording throughout for clarity (shown in the “track changes” version).

**2. Line 151: The RPE is mentioned here for the first time, without explanation or prior definition (abstract doesn’t count). Please fix.**

We have defined RPE within the main text.

**3. Figure captions for figures 3 and 4 say error bars are omitted, even though error bars are now included.**

The figure captions have been modified accordingly.

**4. Around line 534, the authors could acknowledge that the effect of compound type in Experiment 1b is small (in addition to ‘direct’) to cohere better with the results section (which says ‘slightly greater’), and lines 596-97 in the discussion, where potential reasons are given for the small effect size.**

Though the point appears belabored, we have added that the effect is small in line 545.
